# Amyloids and brain cancer: molecular linkages and crossovers

**DOI:** 10.1042/BSR20230489

**Published:** 2023-10-03

**Authors:** Shalini Singh, Vibhuti Joshi, Arun Upadhyay

**Affiliations:** 1 Department of Bioscience and Bioengineering, Indian Institute of Technology Jodhpur, Jheepasani, Jodhpur, Rajasthan 342001, India; 2 Department of Neurology, Northwestern University Feinberg School of Medicine, Chicago, IL 60611, U.S.A.; 3 Department of Biotechnology, School of Engineering and Applied Sciences, Bennett University, Greater Noida, Uttar Pradesh 201310, India

**Keywords:** aging, amyloid, Cancer, glioma, neurodegeneration, proteostasis

## Abstract

Amyloids are high-order proteinaceous formations deposited in both intra- and extracellular spaces. These aggregates have tendencies to deregulate cellular physiology in multiple ways; for example, altered metabolism, mitochondrial dysfunctions, immune modulation, etc. When amyloids are formed in brain tissues, the endpoint often is death of neurons. However, interesting but least understood is a close connection of amyloids with another set of conditions in which brain cells proliferate at an extraordinary rate and form tumor inside brain. Glioblastoma is one such condition. Increasing number of evidence indicate a possible link between amyloid formation and depositions in brain tumors. Several proteins associated with cell cycle regulation and apoptotic pathways themselves have shown to possess high tendencies to form amyloids. Tumor suppressor protein p53 is one prominent example that mutate, oligomerize and form amyloids leading to loss- or gain-of-functions and cause increased cell proliferation and malignancies. In this review article, we present available examples, genetic links and common pathways that indicate that possibly the two distantly placed pathways: amyloid formation and developing cancers in the brain have similarities and are mechanistically intertwined together.

## Introduction

Brain tumor is a widely used term encompassing multiple diseases of heterogeneous nature. Malignant and benign tumors of central nervous system (CNS) include more than hundred distinct pathologies with highly diverse etiology and epidemiology. Despite being less common, brain-associated cancers share disproportionately higher mortality among all cancer types. According to American Cancer Society’s data, more than 300,000 cases of primary CNS (both brain and spinal cord) tumors have been recorded globally in 2020, while approximately 250,000 people died in same year with these tumors. In addition, incidences of secondary brain tumors are also prevalent adding up to these numbers. Secondary brain tumors originate extracranially but travel to and spread inside the brain. Five-year survival rate for brain cancer patients is 36% in United States. Contrary to extracranial tumor types, CNS or brain tumors show specific physiological characteristics owing to the microenvironment present in the brain tissues. The unique cell types, metabolic constraints, and anatomy of the brain present exceptional technological challenges in understanding and developing treatments. One specific feature that brain tumors present is the deposition of amyloid-like formations in the brain tissues harboring the tumors. In 1981, Spaar et al. reported amyloid-like structures in surgically removed brain tumor mass from a forty-six-year-old woman [1]. In following years, many studies further reported evidence of aggregates and amyloid structures of several proteins in the tumor microenvironment.

**Figure 1:**
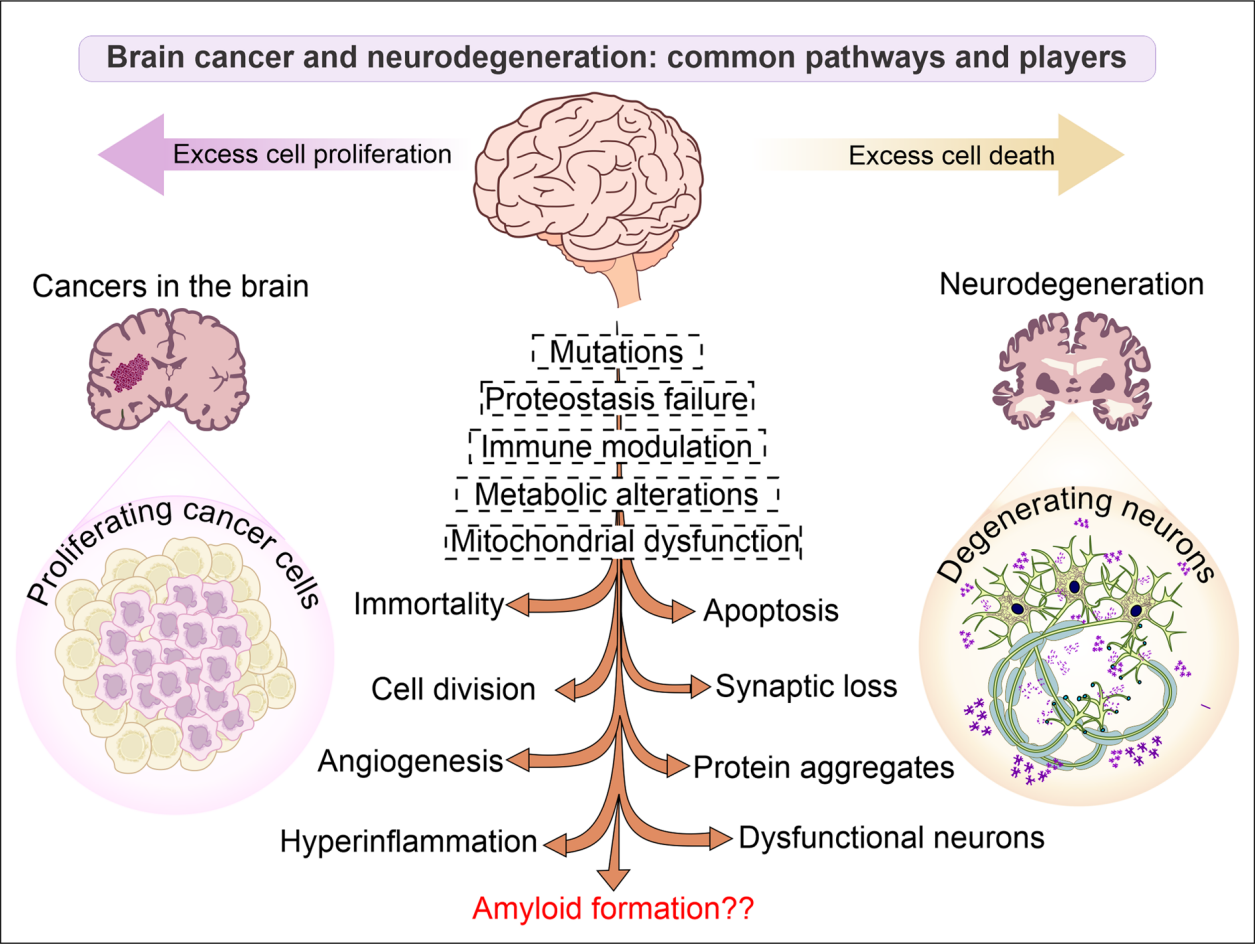
Common links and distinctive features of the two broad classes of diseases: neurodegeneration and brain cancer The two classes of diseases are primarily represented by rapid neuronal loss and unlimited cell proliferation, respectively. There are multiple common pathways affecting systemic changes in these diseases. However, several distinctive features also delineate these diseases and show distinctive characteristics.

Amyloids are one of the most highly studied proteinaceous assemblies, yet our understanding of these structures is not fully grown. In general, several proteins (or peptides) can enter into an amyloid-like state unless prevented by molecular chaperones [2]. These proteins can overburden the cellular proteostasis machinery by forming high-order aggregates inside or outside of the cells. The most studied peptide of this class is amyloid beta 42 (Aβ42), which is responsible for devastating changes happening in the human brain during Alzheimer’s disease (AD), one of the most prevalent neurodegenerative disorders (NDDs). Multiple other proteins can accumulate inside or outside of the brain cells, including neurons and glia, and cause pathological changes in brain tissues. Increased oxidative stress, mitochondrial dysfunction, and neuroinflammatory changes accompany the degenerative changes in many such diseases. Although several pieces of evidence indicate that neurodegeneration and cancers represent two far ends of the same spectrum consisting of multiple pathways in common, but in mutually opposite directions. Nonetheless, a few common links between the two conditions could be identified (Figure 1). The formation of protein aggregates and cells’ inability to clear those deposits are such changes that have been observed in many types of brain tumors. In addition, multiple cell cycle regulatory proteins have previously been identified to form aggregates [3]. In addition, multiple known amyloid proteins have been identified in cancer tissues, including Aβ42, immunoglobulin light-chain amyloidosis (AL), and transthyretin (TTR) [4–6]. However, a clear biological link between amyloid formation and tumor development has not been established so far.

**Figure 2:**
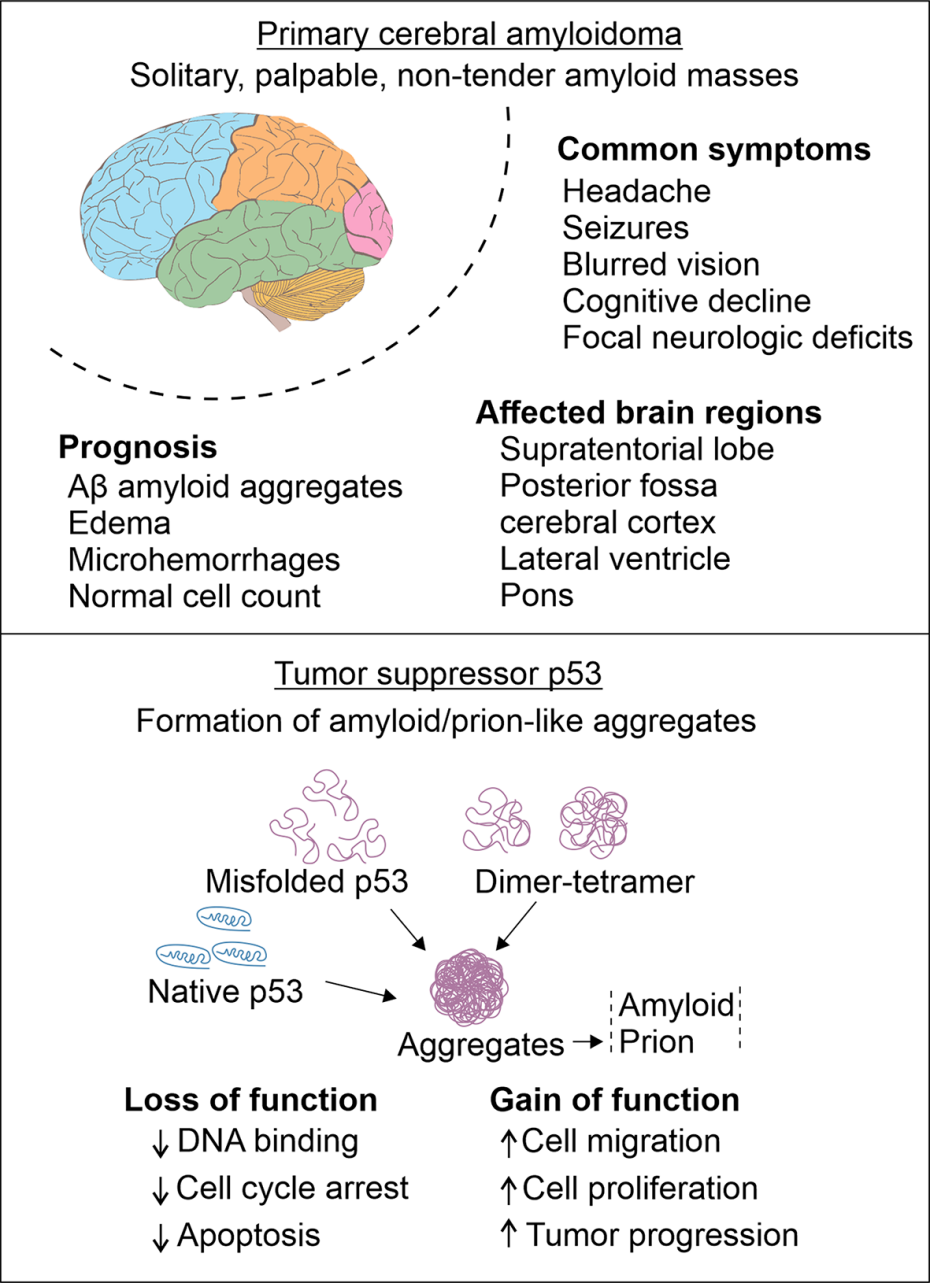
Common link between brain tumors and amyloid formation Primary cerebral amyloidoma is a tumor-like formation that presents both features simultaneously: neoplasia and neurodegeneration although number of cells remain consistent. Another important link between the two conditions is p53 aggregates. Formation of these aggregates leads to loss- or gain-of-functions that lead to poor DNA binding, altered cell cycle progression, apoptotic changes and many others. Several reports indicate p53 aggregates may develop amyloid or prion-like depositions as well, which a common feature of most, if not all, neurodegenerative diseases.

The most common tumor suppressor protein TP53 (p53) has been observed to self-polymerize and accumulate inside the breast and skin cancer cells [7]. As shown in Figure 2, overexpression of this stress-sensing protein followed by cytoplasmic sequestration and oncogenic gain-of-function is observed in undifferentiated neuroblastoma, and glioblastoma [8,9]. The p53 gain of function may also lead to aggregation of p53 paralogs (p63 and p73) into cytoplasmic inclusions, activate stress response pathways and cause transcriptional irregularities [10]. Further investigation of p53 aggregates indicated a prion-like amyloid oligomerization pattern [11]. This aggregation leads to chemoresistance in cancer cells as well, which could be reversed by inhibiting the p53 aggregation [12,13]. In a recent study, when tested independently, an amyloid inhibitor molecule ADH-6 (alcohol dehydrogenase-6) potentially suppressed p53 self-assembly and restored its tumor-suppressor function by inducing cell cycle arrest [14]. Phosphatase and tensin homolog (PTEN), another clinically relevant tumor-suppressor protein, also shows high tendency of aggregation under physiological conditions, which could be a contributing factor for cancer and other PTENopathies [15,16]. Similarly, premelanosome protein (PMEL) is another fibril-forming protein that deposits extracellularly in human biopsy samples. These amyloid fibrils can mechanotransduce transcriptional regulator Yes-associated protein (YAP) that can regulate the melanoma progression by regulating the activities of other genes and affecting the drug resistance in metastatic cells [17]. These examples indicate that amyloid formation from a number of proteins is an important and crucial phenomenon that has been known in other cancer types but not addressed adequately in glioma and other brain malignancies.

A line of accessory factors, other than amyloid deposits, show common origin and progression of the two disease conditions (viz., amyloid formation and cancers) in the brain. For example, presence of a conducive environment in and around the brain tumors can affect multiple proteostasis pathways affecting proteome-wide alterations leading to protein aggregation and amyloid deposition. Several common environmental risk factors that include but are not limited to metal ions like mercury, lead and arsenic; electromagnetic fields; smoking, hair dyes; pesticides; benzene; petroleum products and lubricating oil, etc., also harbor both types of pathological changes [18]. In addition, a number of common genetic factors have been identified that play prominent roles in the etiology of both, brain tumors and the neurodegeneration. Some of the genes are: Inositol polyphosphate-5-phosphatase D (INPP5D), triggering receptor expressed on myeloid Cells 2 (TREM2), Spi-1 proto-oncogene (SPI1), CD33 etc. (Figure 4). We must mention here that despite being several commonalities, a number of genetic factors and molecular pathways that differentiate the etiology and pathological progression of the two types of the above-mentioned diseases. Similarly, enormous metabolic alterations, uncontrolled cell division, angiogenesis and metastasis are some of the hallmarks of cancer tissues. On the other hand, NDDs are majorly exaggerated due to abnormal accumulation of protein aggregates, synaptic dysfunction and uncontrolled neuronal deaths [19,20].

In this article, we are presenting a brief literature on the available common links among the conditions that include, but are not limited to protein aggregation, amyloid formation, brain tumor malignancies and proliferation. We have taken glioma and AD as two most studied cases of the two classes of the disease in question.

## Primary brain tumors: a brief introduction

Tumors are abnormally grown non-functional tissue mass primarily caused due to excessively high rates of cell proliferation. These atypical growths could be both benign or malignant and may eventually turn out to be a pathological lump leading to a number of diseases, including cancers of different organs or tissues. The definition of a primary brain tumor appears very simple as a tumor originating from the brain; however, the complexity is much higher than many other cancer types. Based on the site of origin and histopathology, primary brain tumors may fall into the category of glioma, schwannomas, glioblastoma, pineal region tumor, meningioma, pituitary adenomas, and a few other kinds of tumors [21,22]. The type of primary brain tumor varies largely among adults and pediatric groups, and it has been reported that glioblastoma is the most common metastatic brain tumor among adults [23,24]. Although brain tumor is not an age-related abnormality, elderly patients are the highest among all age groups with a primary brain tumor and present multiple challenges in treatment because of comorbidities [25]. The most common symptoms of primary brain tumors are headache, nausea, double vision, speech difficulty, seizures, and many more [26,27]. Molecular analysis of primary brain tumors suggests that p53, PTEN, isocitrate dehydrogenase (IDH), telomerase reverse transcriptase (TERT), α-thalassemia/mental retardation syndrome X-linked (ATRX), and epithelial growth factor receptor (EGFR) are most commonly mutated genes in these tumors [28].

**Figure 3:**
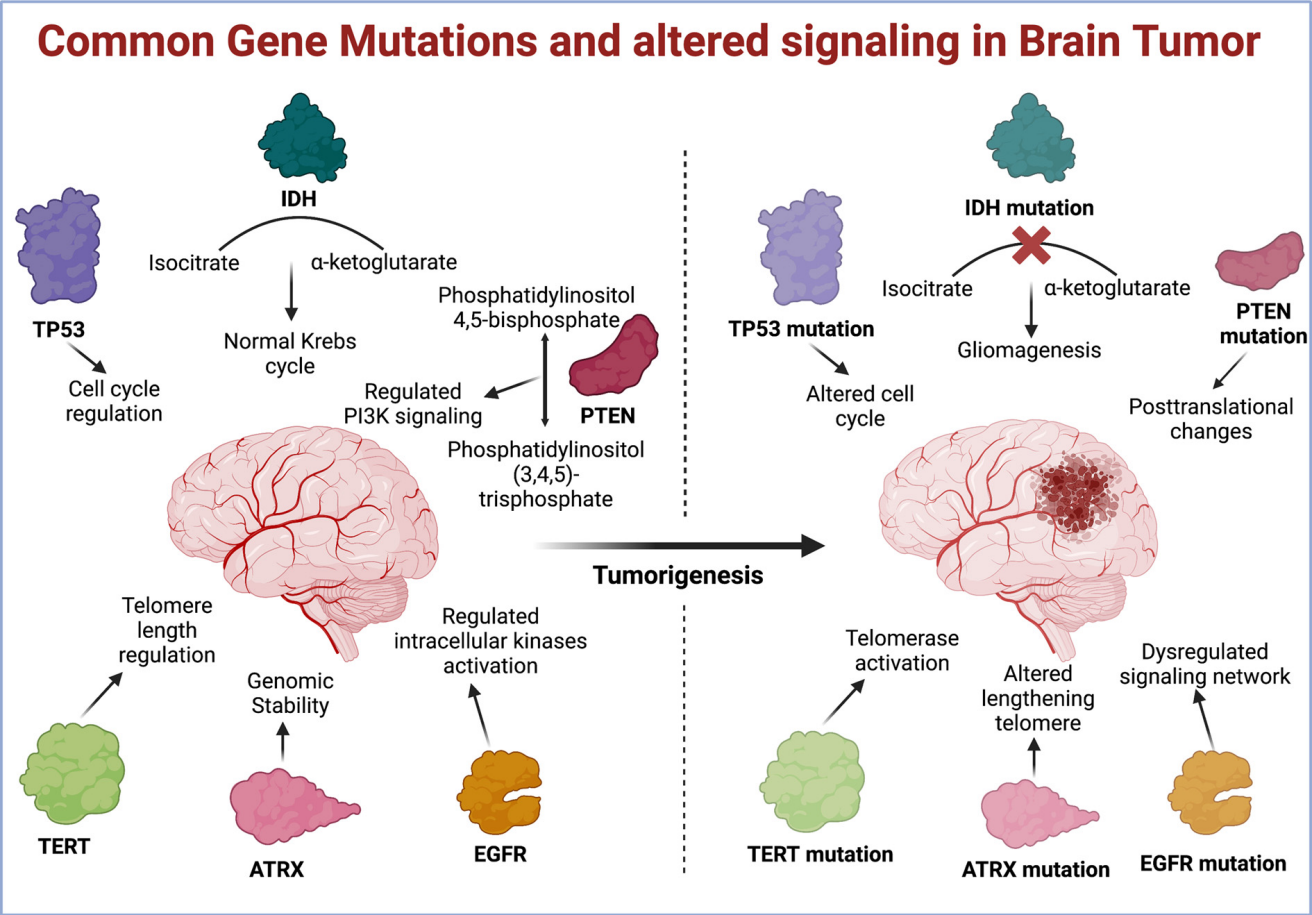
Key genetic mutations in brain tumors Cancers are always an outcome of multiple gene mutations. Some mutations are common for different cancers and some are specific to cancer types. Common genetic mutations in all kinds of brain tumors are summarized in this figure. Each of these gene mutations alters different cellular pathways, which leads to tumorigenesis. The figure was created with Biorender.com.

Among these mutated proteins IDH plays a key role in maintaining cellular homeostasis in tumor cells by catalyzing oxidative decarboxylation of isocitrate in Kreb cycle. Further, the metabolic reprogramming by IDH mutation generates redox imbalance and epigenetic alterations, making this gene a prominent therapeutic target [29,30]. PTEN is another gene in this category, mutation of which leads to abnormal activation of phosphoinositide 3-kinase (PI3K) pathway and determines the severity of brain tumors. Glioma patients with PTEN mutation have shown poor survival and increased drug resistance [31]. As a tumor suppressor and transcription factor, TP53 is the most oldest known gene to be mutated in glioblastoma patients [32]. Apart from forming amyloid fibrils, the mutated p53 also affects multiple other pathways, such as Wnt signaling to develop drug resistance [33]. TERT and ATRX are the other two prognostic markers of glioblastoma [34,35]. TERT, as specialized reverse transcriptase, promotes the addition of hexamers at the 3′-end of chromosome, thus prevents the tumor cells senescence [36]. ATRX mutations, instead affect the chromatin remodeling and genomic stability, and further show a strong correlation with altered length of telomeres [37,38]. EGFR is among the most recent targets for glioblastoma. EGFR mutations cause metabolic reprogramming and affect multiple epigenetic changes in glioma cells [39,40]. In addition to these genes, several other genetic mutations have been identified using whole exome sequencing that underlie molecular pathogenesis of glioma. Many of these are currently under investigation and are studied for drug development [41,42]. In Figure 3, we have summarized the above-mentioned gene mutations and affected pathomechanisms in glioma.

Brain tumor diagnosis mostly relies on magnetic resonance imaging or positron emission tomography [43]. The current point of care therapy for primary brain tumors includes surgery, followed by radio-chemotherapy that only improves the average patient’s survival by 5 years [44]. Unlike other cancers, primary brain tumors are targeted by very few treatment regimes, majorly because of the blood–brain barrier that strongly influences the treatment method [45]. In spite of these aggressive treatment methods, the recurrence of high-grade primary brain tumors is very common among patients, which compels researchers to find novel methods of treatment [46]. Immunotherapies that include but are not limited to checkpoint inhibitors, cellular or viral immunotherapy, and vaccines, are additional methodologies applied toward addressing primary brain tumors [47,48]. Gene therapy, nanomedicine, and phototherapy are the other advanced methods that are under consideration for future treatment regimens for primary brain tumors [49]. As discussed, one important aspect of primary brain tumors is their connection with NDDs; strong molecular crosstalk has been observed between these two classes of disease [50]. NDDs are primarily caused due to formation of intra- or extracellular protein aggregates, amyloids or inclusions, that may affect the proteostasis machinery and leads to death of neurons. Mitochondrial dysfunction, oxidative and proteotoxic stresses, inflammatory changes, and immune modulation are other common pathological changes observed in most NDDs [51–53]. In upcoming sections, we will discuss the links between primary brain tumors and NDDs by discussing examples of several common symptoms and molecular pathways.

## Brain tumors and memory loss: common links and growing evidence

Dementia is one of the common symptoms among brain tumor patients. Moreover, surgical interventions also impact the memory of brain tumor patients [54]. A case study of brain tumor patients reported a significant association with dementia which includes impairment of cognitive activities and loss of memory [55]. One case–control study also reported memory loss as a significant parameter in assessing clinical features of primary brain tumors [56]. In another study, poor working memory has been observed among pediatric brain tumor patients, linking memory loss with primary brain tumor irrespective of the patient’s age [57,58]. Pediatric primary brain tumor survivors also show long-term working memory impairment after radiation and other therapeutic measures [59,60]. Among adult patients, neuropsychological evaluations reported major episodic memory loss with a primary brain tumor and continued even after resection of the tumor [61,62]. Psychosocial distress among primary brain tumor patients adds to memory loss and needs further attention [63]. Another important aspect of memory loss in brain tumor patients is the location of the primary brain tumor. Frontal Lobe tumors directly impact multiple cognitive functions, including memory loss [64]. Recent study reported that 60% of primary brain tumor patients in their study group show some kind of memory loss. Out of them, 47% had recent memory loss, while others had remote memory loss [65].

Malignant brain tumors are well-known for deaths of neurons. The mechanism of neuronal cell death in brain tumors is similar to neurodegenerative disorders, like Alzheimer's disease [66]. It is also known that physical pressure on brain by tumor mass of primary and metastatic brain tumors can induce neuronal cell death and cause loss of functions. In one study, lithium was explored to reduce this solid stress in mice model to control cell death [67]. Another pathway of glioma-induced neuronal cells death has been reported by the increased glutamate excitotoxicity caused by the expression of oncogenes like AEG-1 (astrocytes elevated gene-1). The finding was further confirmed by a negative correlation between the expression of AEG-1 and NeuN [68]. ATF4 (activating transcription factor 4) is another protein that causes neuronal cell death and promote angiogenesis of primary brain tumors [69]. A recent study reported that glioma secretes glutamate in the tumor microenvironment, which also enhances neurodegeneration. The glutamate release would impact peritumoral neurons and cause hyperexcitability in glioma tissue which further lead to excitotoxic cell death [70]. All these studies suggest the strong pathophysiological and molecular common links between the neurodegeneration and brain tumors (Figure 1).

Inactivation of tumor suppressor p53 gene due to mutations is one common factor found in ∼50% glioma patients. Previous studies showed positive impact of virus-mediated p53 gene therapies by markedly reducing the growth of glioma and prolonging the lifespan [71,72]. In a different study, retroviral delivery of p21 and p16 showed better effect than p53 in suppressing the tumor growth [73]. Similarly, another amyloid-forming gene PTEN, when functionally restored in glioma cells, suppressed tumorigenic properties and improved drug sensitivity [74,75]. Apart from tumor suppressor genes and signaling proteins, many cytokines, for example tumor necrosis factor alpha (TNFα), interferons- beta/gamma (IFNβ/γ), and interleukins (IL-12) helped increasing the survival rates by suppressing the tumorigenicity in glioma disease models and human patients [76–79]. It must be noted that these small inflammatory proteins (cytokines and chemokines), lie at the crossroads of molecular pathways involved in tumorigenesis and neurodegeneration; therefore provide effective therapeutic benefits [80,81]. For example, deletion of TNFα, a neurodegenerative cytokine, may help attenuate neuropathological changes [82]. Similarly, chemokine receptor antagonists may also prove to be effective neuroprotective agents [83].

## Amyloid formation in brain tumors

Several tumor types have been found associated with local (within tumor) or systemic (elsewhere) deposition of amyloid aggregates. For example, occurrence of amyloid aggregates of transthyretin, immunoglobulin light-chain, and amyloid beta in breast cancer tissues has been reported in several previous studies [4–6]. Amyloid tumor of the breast, a condition of localized amyloidosis, is one such condition that has been reported in multiple patients in past few decades [84]. Recently, amyloid-specific dyes and antibody-based immunostaining of glioma samples collected from human patients confirmed the presence of Aβ42 peptides and amyloid deposits in astrocytes, close to blood vessels and perivascular spaces [85]. In C6 glioma cells, Aβ42 treatment induces cyclooxygenase-2 and inducible nitric oxide synthase (iNOS) overexpression followed by nuclear factor kappa B (NF-κB), which could be reversed by an anti-aggregatory compound resveratrol [86]. Several other anti-aggregatory natural molecules, for example trehalose, quercetin, and myricetin, have shown anti-neoplastic and apoptosis-inducing potential in glioma cells [87–90]. Another highly effective anti-amyloid agent epigallocatechin-3-gallate (EGCG) leads to cell death, reduced cellular proliferation, and suppression of invasion in multiple glioma cell lines and is suggested to be used as an adjuvant in anti-glioma therapies [91]. These examples indicate several molecular crossovers and provide an opportunity to identify common therapeutic targets and develop effective therapeutic strategies for both classes of diseases. Although our understanding of the two conditions (amylosis and glioma) is limited, several lines of evidence indicate a common lineage or at least some connecting links.

### Primary brain amyloidoma: the classical example

In 1981, Spaar et al. reported deposition of walnut-sized amyloid mass in a patient suffering from progressive visual loss with scotoma and finally from hemianopia, associated with attacks of headaches and recurrent episodes of depression [1]. It consisted of both amyloid deposits and plasma cells. This classic example of primary cerebral amyloidosis characterized by localized tumor-like amyloid deposition was later called as primary brain amyloidoma. These amyloidic deposits of glycoproteins remain benign; however they may mimic malignant brain tumors as evident in a recently reported case in a 65-year-old Caucasian woman [92]. Primary brain amyloidoma is a rare tumor-like lesion, which consists of deposition of amyloid within the brain parenchyma without evidence for systemic amyloidosis. Amyloid accumulation was found exclusively in the white matter of different supratentorial brain regions. Neuropathological analysis revealed the presence of amyloid deposits surrounded by plasma cells, monocytic cells as well as foreign body type cells. Another case, which developed in a sixty-nine years old male, composed of lesions consisting of amyloid AL λ [93]. In the brain, usually neuro-axial and extra-axial masses occur. In another case, in a 26-year-old woman was also represented by lymphocytic and plasma cellular infiltrates [94]. Majority of plasma cells confirmed the presence of lambda light chains and one-fourth of plasma cells also expressed kappa light chains as well. A long-term follow-up described brain amyloidomas as benign with respect to the cellular composition but the variability in the magnetic resonance imaging signal showed the presence of nonuniform progressive amyloid deposits [95]. Although the clinical course of the disease is non-progressive, yet amyloid lesions lead to memory loss and mild cognitive impairment [96]. Another case in a 34-year-old male that progressed into death within 2 years following the diagnosis consisting of monoclonal lambda light chain originated amyloid formation in most of the vessels [97]. The cerebral and cerebellar white matter, basal ganglia, and thalamus were the most affected areas. In a case report done by Lohr et al. in 2019, showed the presence of several inflammatory cell population such as microglia, macrophages, T cells, as well as scattered B cells [98]. Primary brain amyloidoma shows the combination of two different nosological entities consisting of cerebral neoplastic diseases and neurodegeneration. The pathological symptoms of cognitive damage, such as memory loss, cognitive decline and other neurological dysfunctions are the result of inflammation, and oxidative stress (Figure 2). Lohr et al. also concluded a definitive cure to PBA, since the lesions formed are localized as compared with several other cerebral disorders.

### p53 amyloids: where multiple pathways intersect

One most relevant example connecting these two conditions: the cancer and amyloids is the tumor suppressor protein TP53. This p53 protein has been extensively studied for its roles in cell cycle regulation. Mutation in p53 gene is a common factor involved in pathological manifestation of plethora of cancers. For example, M237I mutant of p53 confers chemoresistance to a highly potent chemotherapeutic drug temozolomide in glioblastoma cell lines [99]. Initial studies indicated that an increased p53 expression in Aβ treated cultured neurons and high amyloid precursor protein (APP) in glioma cells are two observations that drive the commonalities between the two disease types [100,101]. Consequently, U251 glioma cells, when treated with transient axonal glycoprotein-1 (TAG1), an APP ligand glycoprotein molecule presented high expression of p53 and its downstream target genes, contributing to increased cell proliferation [102]. In addition, Aβ-mediated toxicity leads to p53-mediated microglial apoptosis; and inhibition of p53 may provide therapeutic benefits in AD [103]. In recent years, a line of studies has indicated that this protein has an inherent amyloid formation tendency. Considering the importance of cell cycle regulatory potential of this gene, its possible conversion into amyloidogenic oligomers has generated enormous interest leading to detailed investigation (Figure 2). One recent study showed that nuclear deposition of p53 amyloids may help gain chemoresistance in glioblastoma-derived cells [9]. Amyloidogenic p53 alters tumor-suppressor functions and induces p53-mediated oncogenic pathways [104]. For example, p53 gain of function mutation leads to deregulated cell cycle, apoptosis, senescence and inflammatory changes affecting the prognosis and treatment outcomes [105,106]. Another study confirmed penetration of preformed p53 aggregates by macropinocytosis process and seeding amyloid-formation in intracellular p53 protein, a tendency of spread very similar to prion proteins [107]. The amyloid-like formations and prion-like transmission mechanism of mutant p53 have been exploited for their therapeutic potential in the past. Gene therapy, RNA interference, peptide stabilizers, and small molecules are the most common tools to target this protein. Unfortunately, most of these approaches could not result in successful prevention of tumor growth and progression [108].

## The genetic links between brain tumors and neurodegeneration

**Figure 4:**
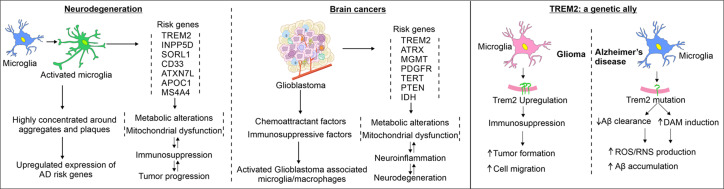
Genetic and immunomodulatory factors among cancers and degenerative diseases in the brain Microglia cell activation is a common factor in both, tumor microenvironment and amyloids nanoenvironment. These cells, through a highly regulated but complex mechanisms, initiate a number of systemwide changes that may impact, alter or worsen the conditions. Several genes have been identified in the past affecting these diseases independently as well as in concerted manner. One common gene, that has been studied in great detail in conditions of brain tumors is TREM2. In recent years, it has been projected as a potential AD gene and has been tested for its potential roles in affecting amyloid formation pathways.

Glioblastoma is often referred as a cold-tumor due to presentation of an immunosuppressive environment. Although the disease can occur in any age group, its higher prevalence in late-age individuals is very common. Aging brain consists of a number of pro-inflammatory changes primarily mediated by several age-associated genetic manipulations happening in the brain [109]. Serpina3 is one such immunomodulator gene that has varying changes in expression at late age. This protein has also been found playing crucial roles in developing brain amyloidosis in AD brains by increasing the Aβ aggregation [110,111]. Contrary to that, Serpina3 gene silencing in rodents leads to suppressed intracranial tumor growth followed by increase in animal survival. In the same study, rescue experiments confirmed that cell migratory behavior in patient-derived GBM cells was found to be dependent on endogenous Serpina3 gene [112]. All these results indicate that age-associated Serpina3 protein could be a possible connecting link between amyloid formation and glioma cell proliferation. Another age-related gene C-C chemokine receptor type 7 (CCR7) deficiency leads to deleterious neurovasculature, activated microglia and amyloid formation in aging brain [113]. This gene can induce transforming growth factor beta 1 (TGF-β) signaling, followed by nuclear factor kappa B (NF-κB) mediated matrix metallopeptidase 2/9 (MMP2/9) overexpression orchestrating migration, invasion and epithelial–mesenchymal transition in malignant glioma cells of human origin [114]. Similarly, stress-induced self-aggregation of TGFβ1-induced anti-apoptotic factor 1 (TIAF1), an upstream regulator of TGF-β/Smad signaling leads to APP degradation and generation of Aβ42 peptides. When injected in nude mice subcutaneously, the U87-MG glioma cells lead to the formation of TIAF1 and Aβ42 amyloids in growing tumor mass and confer drug resistance [115–117]. AD risk genes have been linked directly to the pathways and changes associated with astrocytoma, glioblastoma and other cancers. For example, a transcription factor SPI1 contributes to migration of glioma, while its lowered expression level is linked with a delayed onset of AD [118,119]. Sortilin related receptor 1 (SorL1), a putative AD-linked gene was found downregulated in astrocytoma of high grade [120,121]. Another lipidostasis associated AD risk gene ATP-binding cassette transporter A7 (ABCA7) found elevated in glioma cells [122,123]. Knockdown of microglial protein INPP5D leads to increased plaque formation, while depletion of this gene affects the migration of glioma cells [124,125]. Similarly, one inflammation and metastasis related protein S1004A that acts as signature gene highly expressed in Tregs and exhausted T cells has been shown forming amyloid-like aggregates in multiple pathological conditions [126,127]. In addition, there are multiple other genes or proteins that have been shown to play crucial roles in brain cancer cells as well as in protein aggregation-mediated neurodegeneration (Figure 4). However, discussing all those genes is beyond the scope of this review.

### TREM2 affects genetics of cognitive dysfunction and neuroinflammation

Triggering receptor expressed on myeloid cells-2 (TREM2) is an innate immune receptor which is expressed on the cell membrane of monocyte-derived dendritic cells, osteoclasts, and microglia [128,129]. TREM2 expression is sensitive to different areas of the brain depending upon the variation and localization in the concerned brain pathology. TREM2 expression has been suggested to have protective roles in clearance of apoptotic neurons [130]. TREM2 functions via signaling adaptor protein DNAX-activating protein 12 (DAP12). A mutation in both these proteins was described by two independent studies done by Nasu and Panu Hakola causing Nasu-Hakola disease or polycystic lipomembranous osteodysplasia with sclerosing leukoencephalopathy (PLOSL) [131,132]. PLOSL is described by presence of early dementia and bone cysts with subsequent bone fractures. TREM2 expression is sensitive to different areas of the brain depending upon the variation and localization in the concerned brain pathology. In a study conducted by Stefano et al. in a neuroblastoma cell line N2A expressing HSP60 (heat shock protein family D member 1), the interaction of HSP60 and TREM2 was established. This interaction confirms the bridging between apoptotic neurons and astrocytes with microglia for an effective removal [133]. TREM2 expression has been also associated with aging-related senescence and progressive dementia in mice [134]. In a single cell analysis, functional pathways of AD associated microglial cells have been shown to be partially regulated by TREM2 [135].

TREM2 role has also been suggested to increase with AD-related neurofibrillary pathology in a study conducted on postmortem inferior temporal cortex samples obtained from 60 patients [136]. These patients were divided into three groups with respect to the Braak stages (0-II, II-IV and IV-VI). Paraoxonase 1 (Pon1) is a protein associated with AD, Parkinson's disease and other brain disorders [137]. In a study conducted on Pon1 knockout mice, an increased expression of TREM2 led to the increased microglial phagocytosis and lowered the level of pro-inflammatory cytokines. HIV (human immunodeficiency virus) associated cognitive disorder is also associated with the expression and functional role of TREM2 [138]. In a study conducted on brain tissues of 52 HIV-positive patients diagnosed with relative cognitive disorders had altered expression of TREM2 and TNFα. The soluble fraction of frontal cortex was increased as compared to the functional membrane bound fraction suggesting an impaired TREM2 protein function. Altered expression of TREM2 and Aβ-associated inflammation was more prevalent in HIV positive Hispanic patients as compared to non-Hispanic cohort. In conclusion, TREM2 plays an important role in clearance of dead neurons and protects against neurodegeneration in various cerebral disorders (Figure 4).

TREM2 have different roles in various pathologies, cancer and cell types. For example, substantial evidence confirm a prominent immunosuppressive impact of TREM2 in cancer cells. The cell-specific TREM2 functions, such as in fibroblast and stromal cells are different than the actual tumor microenvironment [139]. Deficiency or absence of TREM2 enhances the effects of therapeutic drugs and restrains overall tumor growth [140]. TREM2 promotes T cell dysfunction and immunosuppressive activity of myeloid cells in tumor microenvironment. Ablation of Trem2 gene may suppress Mreg, exhausted CD8^+^ T cells and thus, the overall tumor growth [141]. On the other hand, another independent study showed that use of anti-TREM2 monoclonal antibody modulated and suppressed the tumor-associated macrophage population and also led to the infiltration of CD8^+^ tumor associated lymphocytes in tumor microenvironment [142]. Another extensive study based on the mouse model of Trem2^–/–^ and wildtype mice in 3-methylcholanthrene-induced sarcoma, colorectal cancer and mammary tumor showed that anti-PD-1 immunotherapy is more effective in Trem2 deficient mice as compared to wild type mice. Anti-TREM2 monoclonal antibody treatment prior to PD-1 immunotherapy that made tumors more susceptible and increase the efficacy of the former drug in different cancer types, including glioblastoma [140,143]. It was also suggested that TREM2 expression led to the inhibition of metastasis and downregulated the extracellular signal-regulated kinase (ERK) pathways.

## Neuroinflammation reconnects pathways of cancer, neurodegeneration and aging

Neuroinflammation is defined as a complex inflammatory response in the brain and other parts of the central nervous system in response to injury, infection, stress, neurogenerative diseases, age, and cancer [144]. Release of chemokines, cytokines, and reactive oxygen species are the key characteristics of neuroinflammation, moreover, impairment of cognitive activities is generally linked with its long-term effects [81,145]. Neuroinflammation could be good as well as bad. An example of good inflammation is the case of trauma, when inflammation helps induce healing and repair pathways. On the other hand, it may also accelerate aging pathways. Hyperactivation of neuroinflammation leads to neurodegenerative diseases, like multiple sclerosis while suppression of such response helps primary brain tumors to survive and grow further [146,147]. In fact, neuroinflammation is associated with both neurodegeneration and primary brain tumors and may have contrasting effects on cancer and neurodegenerative pathways [148,149]. Notably, the mechanism of activation of CNS inflammatory responses is a highly complex and tightly regulated process [150]. Brain immune cells microglia and astrocytes are the major players in the neuroinflammatory response of CNS [151]. Microglia, a parenchymal cell type, constitute 10% of brain cell population and perform highly specialized functions. Roles of these cells in mediating immunological responses and causing neurodegenerative changes have gained high attention in recent years [152]. Astrocytes are more common among brain cells and show distinct functions, like regulating flow of blood, neurotrophins and metabolites. They also help maintain integrity of blood brain barriers. A combination of signals of these two cells constitute the overall immune response inside brains, For example M1 microglia and A1 astrocytes are primarily neurotoxic, while M2 microglia and A2 astrocytes may have protective functions in the brain. The immune responses are highly complex and need more research to provide a mechanistic distinction under various diseases [149].

As mentioned, brain tumors are primarily known for their immune-suppressive tumor microenvironment; but generate a neuroinflammatory response within tumors that supports tumor metastasis and invasion [153,154]. Neuroinflammation in brain tumors could be associated with necrosis and new vascularization, thus promoting migration, proliferation, and angiogenesis of tumor cells [155]. Rather than generating a strong anti-tumor response, inflammation supports tumor growth and progression [156,157]. Cytokines' pattern in neuroinflammation decides the direction of the immune response, such as the release of proinflammatory mediators like cytokines, free radicals, chemokines, and matrix metalloproteins (MMPs), which helps in cancer initiation and metastasis [158]. Another association of neuroinflammation with brain tumors has been reported after radiation therapy, which further impacts the cognitive abilities of the patients [159,160]. Scientists also studied the connection between neuroinflammatory markers and brain tumor types to target neuroinflammation for brain cancer treatment [161]. Case study of 35 patients with glioma brain tumors reported that neuroinflammatory markers, like procalcitonin and C-reactive protein, are prognosis markers of glioma, further confirming the connection between neuroinflammation and brain tumor [162]. Michael Heneka group has shown the positive effect of knocking down one of the most crucial inflammatory protein Nlrp3 from the AD mice. The knockdown resulted in no plaque formation and improved memory and behavioral deficits [163]. A recent study by Heneka group showed a phenomenon called ‘a perfect storm’ caused by amyloid cross-seeding mediated by microglial origin ASC (apoptosis-associated speck-like protein containing A CARD) specks [164]. Despite collecting evidence of a clear connection between neuroinflammation and brain tumor, there are multiple underlying mechanisms that are not explored so far and need to be studied in depth. Neuroinflammation is often accompanied by plethora of metabolic reprogramming in both neurons and glia (Figure 4). Such bioenergetic manipulations are common in several NDDs. Unfortunately, our understanding of complex neuroimmunometabolic homeostasis pathways is distorted and needs more detailed studies.

## Conclusions and future directions

In his initial drawings of affected AD brain regions, Dr. Alois Alzheimer has depicted glial cells huddle around the degenerating neurons. Now, after a century of research, we know with certainty that inflammation plays very important role in the mediating neurodegeneration or *vice versa*. Several common factors, including environmental, and genetics have been identified so far that can induce one of the two and end up in exaggerated deterioration caused by the other pathway complementing the first one. Recent studies suggest that plaque surrounding microglia are highly specialized cells, harboring a distinct subset of proteome compared to other microglial cells. These specialized cells try to combat the spread of the plaque by inducing damage-sensing pathway gene networks. In addition, the cells fine-tune their housekeeping and tolerogenic functions in order to slow down the loss of neurons by eating more of defective molecules [135,165]. Trem2 and ApoE (Apolipoprotein E) play essential roles in programming these changes effectively. While ApoE is one high risk AD gene, Trem2 in recent years has been linked with pathogenesis of multiple NDDs. Similarly, amyloidogenic proteins, such as p53, γ-synuclein and tau are involved in both cancer and NDDs. Amyloid evolvability in cancer is defined as the epigenetic inheritance acquired during various stress conditions, such as hypoxia, inflammation and oxidative stress [166]. Both p53 and γ-synuclein have the propensity to oligomerization into protofibrils which provide resistance against these stressors. This resistance can further get transferred to progeny tumors in a prion-like transmission [167]. The commonality between age-associated cancer and NDDs can be further understood by a better understanding of evolvability. In past century, a line of evidence confirmed a close association of amyloid formation pathways with plethora of diseases, which are not limited to neurodegeneration only. Brain malignancies although seem distant from neurodegenerative changes in terms of cell division and proliferation, yet multiple factors confirm a close association of brain tumors with several conditions of amyloid formation. These factors include but are not limited to environment, genetics, immune responses and neuroinflammation, etc. The amyloid formation, neuroinflammation and proteostasis pathways have been investigated thoroughly in past two decades in different animal models and disease conditions related to neurodegeneration and brain tumors.

Several attempts have been made to exploit the close connections (crossovers) among the diseases belonging to the two classes: neurodegeneration and cancers. For example, some therapeutic success has been recorded in terms of overcoming cell malignancies and controlling the tumor growth when subjected to anti-amyloid compounds. Major compounds that showed tremendous success in providing therapeutic benefits in the two diseases are: resveratrol, trehalose, myricetin, quercetin, EGCG, rapamycin, etc. [168–170]. Myricetin, a flavonoid shows anti-migratory and antiinvasive properties against GBM cells [171]. The same compound reduces cytotoxic protein aggregates by modulating the protein degradation potential of 26S proteasome [168]. Some of these compounds show both anti-aggregatory profiles, and also provide neuroprotection in several other ways. For example, they may help in reducing proteotoxic or oxidative stress, enhancing chaperoning capacity of the cells, modulating neuroinflammation, and regulating apoptotic pathways. Several drugs could be repurposed for therapeutic benefits in these two diverse classes of diseases. For example, multiple non-steroidal anti-inflammatory drugs have shown prominent benefits in ameliorating pathological symptoms in different tumorigenic pathways as well as neurodegeneration [172–174]. As summarized in Table 1, all these examples indicate that despite completely distinct pathological manifestations, two broad classes of human disorders share multiple common factors, including causative mechanisms, affected pathways and possible therapeutic solutions. However, our knowledge of how to target these diseases, by exploiting common genetic factors, crucial protein players and by using small molecules is fragmented.

**Table 1:** Literature evidence summarizing common characteristics, genes, possible pharmacological interventions in brain cancers and neurodegenerative diseases

**Clinical conditions/pathological features showing possible crossovers**		
Primary brain amyloidoma	Amyloid deposits with tumor like appearance	[1]
Glioma multiforme	Amyloid beta deposits in human glioblastomas	[85]
**Cell types, and their roles in glioma and neurodegeneration**		
Microglia	Mediates neuroinflammation in both; supports glioma progression; involved in uptake and clearance of amyloid	[175,176]
Astrocytes	Regulates transcriptional reprograming in glioblastoma microenvironment; acquire astrogliosis, initiates inflammation and neural damage	[177,178]
Oligodendrocytes	Oligodendrocyte precursor cells (OPCs) may transform and initiate gliomagenesis; functional loss in AD leads to demyelination and increased amyloid pathology	[179,180]
Dendritic cells	Potential use in vaccination/immune therapy of brain cancers; affects anatomical alterations in aging brain	[181,182]
**Common affected molecular pathways in brain cancer and neurodegeneration**		
Proteostasis failure	NDD: increased protein aggregation	[183]
	Cancer: perturbed cellular stress regulation	[184]
Cell metabolism	NDD: wide metabolic alterations	[185]
	Cancer: metabolic reprogramming	[186]
Mitochondrial dysfunction	NDD: increased oxidative stress	[187]
	Cancer: compromized cell death pathways	[188]
Cell proliferation/apoptosis	NDD: high neuronal cell death	[189]
	Cancer: uncontrolled cell proliferation	[190]
Immunity/inflammation	NDD: hyperactivated inflammatory responses	[191]
	Cancer: suppressed immune system	[153]
**Genes affecting both, NDDs and brain cancers**		
Trem2	A glioma risk gene; variant R47H increases susceptibility towards NDDs	[192]
p53	A tumor suppressor proteins; forms oligomers and fibrils in AD brains	[193]
PTEN	A tumor suppressor protein; accumulation in AD brain neurofibrillary tangles	[194]
Apolipoprotein E	An AD genetic risk factor; facilitates lipid delivery to glioma cells	[195]
Cytokines- TNFα, IFN-α,β,γ	Regulators of neuroinflammation, crucial for progression of both brain cancers and NDDs	[81,196]
**Small molecules/drugs with therapeutic potential against NDDs, and brain cancers**		
Resveratrol	Induces autophagy, clears protein aggregates; suppresses tumor growth by p53 activation	[197,198]
EGCG	Impedes protein aggregation, inhibits glioma stem-like cells	[199,200]
Trehalose	Potent autophagy inducer, activates micropinocytosis in glioma cells	[87,201]
Myricetin	Enhances proteasome activity, induces mitochondria-mediated apoptosis in glioma	[168,202]
Quercetin	Induces protective autophagy; antagonizes invasion and anfiogenesis in glioma cells	[203,204]
Rapamycin	Autophagy inducer, inhibits growth in GBM patient-derived cells	[205,206]

Despite the link between brain malignancies and memory loss is slippery, efforts are ongoing to understand this dynamic relation between the aggregation and tumor formation. The clinical interest has gone up in recent years, but we still don't know if enhancing the proteostasis pathways or suppressing neuroinflammation would benefit in neurodegeneration. Similarly, our knowledge about modulation of proteostasis pathways and their impact on cancer progression is elusive. In past years, multiple studies showed that small anti-amyloid drug candidates show high potential for inducing apoptosis, inhibition of tumor growth and malignancies. Contrary to these examples, not many anti-cancerous drug molecules show similar effects in preventing amyloid formation and neurodegeneration. In addition, there are multiple limitations to these studies as well. First, we do not have mouse models that can truly recapitulate AD pathology. Second, it is very difficult to find right patient subjects for testing new drug candidates. In conclusion, neurodegeneration (by amyloid formation) and brain cancers (e.g. glioma) seem to be two pathological conditions that are physiologically opposite and are distantly placed on the scale of cell cycle progression and cell death. However, common molecular signatures, cellular pathways and tissue-specific changes affect both conditions and indicate a close association between two. Future studies investigating the system wide changes may provide more conclusive evidences and reconnect the missing dots.

## Abbreviations

Aβ42: amyloid β42
AD: Alzheimer’s disease
EGCG: epigallocatechin-3-gallate
iNOS: inducible nitric oxide synthase
NDD: neurodegenerative disorder
NF-κB: nuclear factor kappa
OPC: oligodendrocyte precursor cell
PLOSL: polycystic lipomembranous osteodysplasia with sclerosing leukoencephalopathy
